# *Xylopsora canopeorum* (Umbilicariaceae), a new lichen species from the canopy of *Sequoia sempervirens*

**DOI:** 10.3897/mycokeys.30.22271

**Published:** 2018-01-31

**Authors:** Mika Bendiksby, Rikke Reese Næsborg, Einar Timdal

**Affiliations:** 1 NTNU University Museum, Norwegian University of Science and Technology, NO-7491 Trondheim, Norway; 2 Save the Redwoods League, 111 Sutter Street, 11th Floor, San Francisco, CA 94104, USA; 3 Natural History Museum, University of Oslo, NO-0318 Oslo, Norway

**Keywords:** California, epiphytic, *Hypocenomyce*, integrative taxonomy, morphology, multiple DNA sequence alignment, phylogeny, redwood forest, TLC

## Abstract

*Xylopsora canopeorum* Timdal, Reese Næsborg & Bendiksby is described as a new species occupying the crowns of large *Sequoia sempervirens* trees in California, USA. The new species is supported by morphology, anatomy, secondary chemistry and DNA sequence data. While similar in external appearance to *X. friesii*, it is distinguished by forming smaller, partly coralloid squamules, by the occurrence of soralia and, in some specimens, by the presence of thamnolic acid in addition to friesiic acid in the thallus. Molecular phylogenetic results are based on nuclear (ITS and LSU) as well as mitochondrial (SSU) ribosomal DNA sequence alignments. Phylogenetic hypotheses obtained using Bayesian Inference, Maximum Likelihood and Maximum Parsimony all support *X. canopeorum* as a distinct evolutionary lineage belonging to the *X. caradocensis*–*X. friesii* clade.

## Introduction

The squamulose lichen genus *Xylopsora* Bendiksby & Timdal consists of two species, *X. caradocensis* (Nyl.) Bendiksby & Timdal and *X. friesii* (Ach.) Bendiksby & Timdal. The two species were formerly placed in *Hypocenomyce* M. Choisy and referred to as the *H. friesii* group (Timdal 1984, 2001) until Bendiksby and Timdal (2013) showed that *Hypocenomyce* was highly polyphyletic. *Xylopsora* is the phylogenetic sister of the clade consisting of the two foliose genera *Lasallia* Mérat and *Umbilicaria* Hoffm. Those three genera make up the sister clade of the genus *Fulgidea* Bendiksby & Timdal, another *Hypocenomyce* segregate. The four genera together constitute the Umbilicariaceae (Bendiksby and Timdal 2013). *Fulgidea* consists of two species, *F. oligospora* (Timdal) Bendiksby & Timdal and *F. sierrae* (Timdal) Bendiksby & Timdal. *Fulgidea* and *Xylopsora* are morphologically, anatomically and ecologically very similar and differ mainly in secondary chemistry; alectorialic acid and thamnolic acid occur in the former, friesiic acid (= “friesii unknown”) in the latter (Timdal 2001, 2002). All species of *Fulgidea* and *Xylopsora* grow on bark and wood and, with the exception of *X. caradocensis*, show preference for burnt stumps and trunks of conifers.

Coast redwood (*Sequoia sempervirens*) forests are an important component of California’s ecosystems. Spanning more than six degrees of latitude along the Pacific coast (Van Pelt 2001) and containing individual trees that can live for more than 2000 years (Rocky Mountain Tree Ring Research 2017), these forests provide important habitats for many terrestrial species (Sawyer et al. 1999). However, biodiversity occupying the redwood forest canopies remains relatively under-explored because access into the tree crowns, which often grow to over 100 m in height, is challenging. The epiphytic lichen flora in old-growth redwood forests appears to be particularly species rich; an epiphyte survey of just nine large redwood trees yielded 137 lichen species including a new species of *Calicium* (Williams and Sillett 2007, Williams and Tibell 2008).

Recent epiphyte surveys in the crowns of additional large coast redwood trees in the southern part of the geographic range (Reese Næsborg 2017) revealed a previously undescribed species of *Xylopsora*. Here the authors have provided detailed morphologic, anatomic, chemical and molecular description of this new species, as well as characterising the habitat and substrates it occupies.

Establishing a multiple DNA sequence alignment (MSA) of non-coding loci, which often have unequal lengths due to indels, can be both time-consuming and highly subjective with regard to structural correctness. There has been great activity in recent years in the development of multiple sequence alignment tools (reviewed by Kamena and Notredame 2009). Moreover, there is no single recommendation as to what phylogenetic algorithm to use to transform the MSA into a reliable phylogenetic hypothesis. The current, moderately sized dataset has been used to test whether a less time-consuming and more objective approach, SATé-II (Liu et al. 2012), provides similar and meaningful results. Both manual and automatic approaches have been used to establish a concatenated MSA of three loci, of which one is non-coding and highly variable (nrITS). Different methods representing three classes of phylogenetic inference (Bayesian, likelihood and parsimony) have also been used.

## Material and methods

### The specimens

Five specimens of an unknown *Xylopsora* species were collected from *Sequoia sempervirens* trees in the southern part of the geographic range of coast redwood. The new species was documented on five trees in Big Basin Redwoods State Park, Santa Cruz County and on another five trees in Armstrong Redwoods State Natural Reserve, Sonoma County, California. The morphology, anatomy, chemistry and DNA sequences of these newly collected specimens have been studied and then compared to existing descriptions of *Xylopsora* and relatives (Bendiksby and Timdal 2013). A total of 50 accessions (Umbilicariales + Fuscideaceae) and their respective DNA sequences were re-used from Bendiksby and Timdal (2013). Vouchers of the newly collected specimens are deposited at JEPS, NY and O.

### Anatomy

Microscope sections were cut on a freezing microtome and mounted in water, 10 % KOH (K), 50 % HNO_3_ (N), lactophenol cotton blue and a modified Lugol’s solution in which water was replaced by 50 % lactic acid. Amyloid reactions were observed in the modified Lugol’s solution after pretreatment in K. Ascospore measurements are given as X ± 1.5×SD, rounded to 0.5 μm, where X is the arithmetic mean and SD the standard deviation.

### Secondary chemistry

Thin-layer chromatography (TLC) was performed in accordance with the methods of Culberson (1972), modified by Menlove (1974) and Culberson and Johnson (1982).

### DNA extraction, PCR, and sequencing

DNA was extracted from the apothecia of four of the five newly collected specimens. The DNA extraction, PCR amplification (nrITS and mtSSU), PCR product purification, cycle sequencing and DNA sequence assembly and editing were performed as described by Bendiksby and Timdal (2013), including a subset of the oligonucleotide primers used (i.e. the forward primers ITS5 and mtSSU1 and the reverse primers ITS4 and mtSSU3R). The four DNA isolates were deposited in the DNA collection at O (Natural History Museum, University of Oslo).

### DNA sequence analysis

The newly produced DNA sequences (mtSSU and nrITS) were aligned manually using BioEdit 7.2.3 (Hall 1999) into a trimmed version of the DNA sequence alignments used by Bendiksby and Timdal (2013). The resultant concatenated alignment comprised three genetic regions (nrLSU, mtSSU and nrITS) and a subset of 54 accessions representing the Elixiaceae, the Fuscideaceae, the Ophioparmaceae and the Umbilicariaceae. In addition to this alignment, hereafter referred to as “MSAmanual”, the software SATé-II version 2.2.7 (Liu et al. 2012) was also used to establish an automated alignment, referred to as “MSAsate”. Both alignments were analysed phylogenetically using Bayesian Inference (BI), Maximum Likelihood (ML) and Maximum Parsimony (MP) algorithms. The outgroup was defined as a clade consisting of two accessions representing the Fuscideaceae, which were also used for rooting. SeqState v.1.36 (Müller 2005) was used to convert alignments between different formats and FigTree 1.4.0 (Rambout 2006‒2012) for visualising and editing output trees.

The BI analyses were performed as described in Bendiksby and Timdal (2013), but with only six million generations due to the smaller dataset. All trees saved prior to the point where the average standard deviation of split frequencies (ASDSF) fell below 0.01 were discarded as burn-in. For the sake of comparability of results, the evolutionary models GTR+G were used for both loci in the BI analysis (only a limited number of evolutionary models are available in SATé-II).

The software SATé-II simultaneously estimates multiple sequence alignments and ML phylogenetic trees. Prior to analyses, MSAmanual was divided into non-orphan (no empty sequences), single-locus datasets and were de-aligned (i.e. all gaps deleted). The MSAsate and its corresponding ML tree were estimated as a multilocus dataset in SATé-II using MAFFT (Katoh et al. 2005, Katoh and Toh 2008) as the aligner, MUSCLE (Edgar 2004a, b) as the merger and RAxML v.7.2.8 (Stamatakis 2006) as the tree estimator with the GTRCAT model. The limit of iterations was set to 50 and otherwise default settings were used. For comparison, MSAmanual was also analysed phylogenetically using SATé-II under the same settings.

For the MP analyses, NONA (Goloboff 1999) was used in combination with WinClada 1.0 (Nixon 1999), applying the heuristic search option with 2000 replicates and maxtrees set to 10000 and otherwise default settings. Parsimony jack-knifing (JK; Farris et al. 1996) with 2000 replicates was performed and otherwise default setting. Parsimony jackknifing was also performed on single-locus datasets for assessing potential gene-tree incongruence prior to estimating phylogenetic hypotheses based on all three loci.

## Results

**Figure 1.**
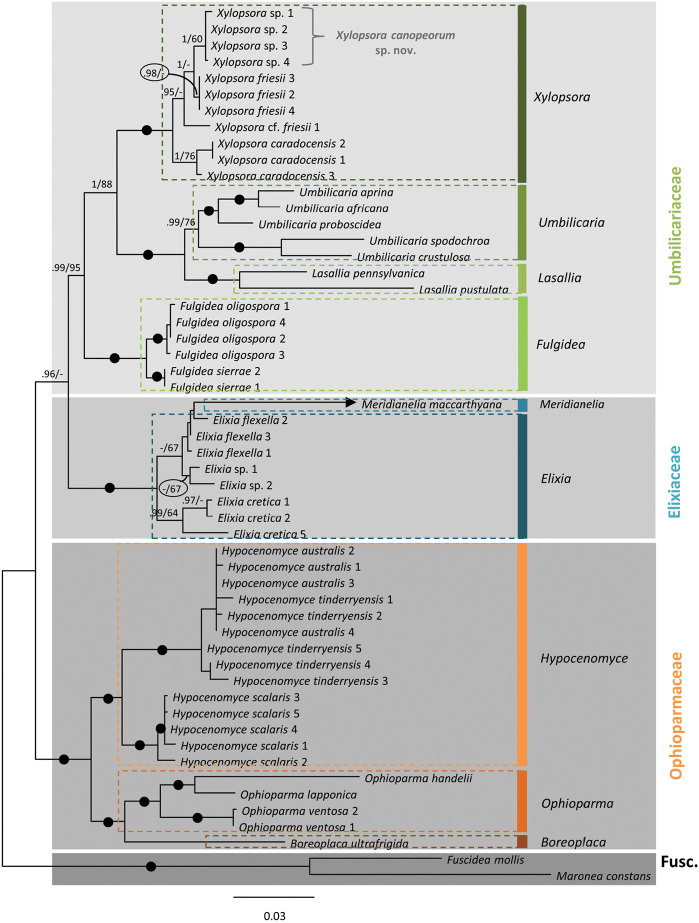
Hypothesis of the phylogenetic relationships and placement of the potentially undescribed species of *Xylopsora* based on DNA sequence data. The depicted topology is based on an automated alignment (MSAsate) of two nuclear (ITS and LSU) and one mitochondrial (SSU) ribosomal loci and is the “best tree” from a RAxML analysis using SATé-II. Clade support over certain values from Bayesian inference (posterior probability; PP) and parsimony jackknifing (JK) analyses are superimposed: PP >0.9 and JK>60% (PP/JK). Clades receiving maximum PP support (1.0) and at least 90% JK support are indicated with a black dot. Multiple accessions of the same taxon are numbered according to Table 1 (also corresponding to the numbering in Bendiksby & Timdal 2013). Family and genus circumscriptions are indicated. Abbreviation: Fusc. = Fuscideaceae. One accession in the tree (*Meridianelia maccarthyana*) appeared on a very long branch that is manually shortened (arrow tipped) to reduce the size of a broad figure.

Four nrITS and three mtSSU sequences were generated (GenBank accession numbers MG309307–MG309313; Table 1). Preliminary parsimony jack-knife analyses of the individual three loci, regardless of the alignment approach, produced congruent gene-trees that were resolved to various extents (not shown). In subsequent analyses, the three loci were analysed in concert. The MSAmanual alignment (i.e. three loci, manually aligned) was 10 characters shorter than MSAsate (i.e. three loci, automatically aligned) and had 12 fewer parsimony informative characters (PIC; Table 2). Both alignments are provided as Suppl. material 1, 2 (MSAmanual.nex, MSAsate.nex). The MSAmanual dataset produced 1220 most parsimonious trees (MPTs) of length 1479, whereas MSAsate produced 10 MPTs of length 1485. Homoplasy measures (Farris 1989) differ negligibly between the two (RC: 46.6 vs 46).The likelihood scores from the RAxML analyses in SATé-II were very similar (Table 2). The ASDSF fell below 0.01 faster in the BI analysis of MSAsate (at generation 820) than in the BI analysis of MSAmanual (around generation 1300). All significantly supported clades were congruent amongst the BI, ML and MP analyses, regardless of the dataset analysed (MSAmanual vs MSAsate). Only results from analyses of the MSAsate dataset are shown (Fig. 1). The authors regarded clade support of at least 60% jack-knife (JK) and at least 0.9 posterior probability (PP) as significant.

**Table 1.** Specimens used in this study with voucher information, major lichen substances, and GenBank accession numbers.

Taxon, Specimen	Voucher Information	Major Lichen Substances	GenBank Accession Number		
			ITS	LSU	mtSSU
Boreoplaca ultrafrigida*	(1) Russia, Sakha Rep., *Haugan & Timdal* YAK03/84 (O-L-138395; ITS). (2) YAK03/39 (O L-127, holotype; LSU, mtSSU)	lecanoric acid	HM161512	AY853360	AY853312
*Elixia cretica* 1	Australia, New South Wales, *Streimann & Curnow* 50968 p.p. (CANB 9304299 p.p.)	–	KF360371	KF360448	–
*Elixia cretica* 2	Mexico, Chihuahua, *Timdal* SON78/03 (O L-15969)	none	KF360372	KF360449	KF360419
*Elixia cretica* 3	Greece, *Spribille* 13340 (GZU, holotype)	–	–	–	GQ892058
*Elixia flexella* 1	Austria, *Halda, Palice & Steinova* 12407 (O L-157191)	–	KF360373	KF360450	KF360420
*Elixia flexella* 2	Turkey, *Palice* s.n. (hb. Palice)	–	–	AY853368	AY853320
*Elixia flexella* 3	*Palice* (ESS 21517)	–	–	AY300837	AY300887
*Elixia* sp. 1	U.S.A., Arizona, *Nash III* 11177 (ASU)	none	KF360374	KF360451	–
*Elixia* sp. 2	U.S.A., Arizona, *Nash III* 41750 (ASU)	none	KF360375	KF360452	–
*Fulgidea oligospora* 1	U.S.A., Arizona, *Nash III* 42735a (O L-767; holotype)	thamnolic acid	KF360395	KF360465	-
*Fulgidea oligospora* 2	U.S.A., Arizona, *Rui & Timdal* US215/01 (O L-59862)	alectorialic acid	KF360396	KF360466	KF360434
*Fulgidea oligospora* 3	U.S.A., Arizona, *Rui & Timdal* US272/01 (O L-59992)	alectorialic acid, thamnolic acid	KF360397	KF360467	KF360435
*Fulgidea oligospora* 4	Russia, Sakha Rep., *Haugan & Timdal* YAK04/05 (O L-18713)	alectorialic acid, thamnolic acid	KF360398	KF360468	-
*Fulgidea sierrae* 1	U.S.A., California, *Rui & Timdal* US249/01 (O L-59964)	alectorialic acid, thamnolic acid	KF360402	KF360471	KF360437
*Fulgidea sierrae* 2	U.S.A., California, *Timdal* SON125/01 (O L-60059; holotype)	alectorialic acid, thamnolic acid	KF360403	–	-
*Fuscidea mollis*	Sweden, *Ihlen* 1372 (UPS)	–	–	AY853369	AY853321
*Hypocenomyce australis* 1	Australia, Australian Capital Territory, *Elix* 19801 (O L-144372)	lecanoric acid	KF360380	–	–
*Hypocenomyce australis* 2	Australia, Victoria, *Krog* Au14/2 (O L-144373)	–	**	–	–
*Hypocenomyce australis* 3	Australia, Australian Capital Territory, *Weber & McVean* s.n. (O L-201, isotype)	lecanoric acid	KF360381	–	–
*Hypocenomyce australis* 4	Australia, Victoria, *Thor* 6047a (S)	–	KF360382	–	–
*Hypocenomyce scalaris* 1	Norway, *Timdal* 11022 (O L-158534)	–	KF360401	KF360470	KF360436
*Hypocenomyce scalaris* 2	U.S.A., North Carolina, *Amtoft* 2058 (DUKE 47763)	–	DQ782852	DQ782914	DQ912274
*Hypocenomyce scalaris* 3	France, *Miadlikowska & Gueidan* 05/24/04-7 (DUKE 47529)	–	HQ650632	DQ986748	DQ986861
*Hypocenomyce scalaris* 4	Sweden, *Wedin* 7141 (UPS)	–	–	AY853373	AY853325
*Hypocenomyce scalaris* 5	Sweden, *Wedin* 7008 (UPS)	–	–	AY853374	AY853326
*Hypocenomyce tinderryensis* 1	Australia, Western Australia, *Elix* 38733 (CANB-790800)	–	KF360407	–	KF360440
*Hypocenomyce tinderryensis* 2	Australia, Australian Capital Territory, *Elix* 33386 (CANB-9801742.1)	–	KF360408	–	–
*Hypocenomyce tinderryensis* 3	Australia, Australian Capital Territory, *Elix* 33387 (CANB-676257)	–	KF360409	–	–
*Hypocenomyce tinderryensis* 4	Australia, New South Wales, *Streimann & Curnow* 50968 (CANB 9304299, holotype)	–	KF360410	–	–
*Hypocenomyce tinderryensis* 5	Australia, Australian Capital Territory, *Streimann & Curnow* 35001 (CANB 610213.1)	–	**	–	–
*Lasallia pennsylvanica*	U.S.A., *Culberson* 22287 (DUKE)	–	HM161513	AF356665	AY631278
*Lasallia pustulata*	Norway, *Hestmark* 3202 (DUKE 47908)	–	HM161456	DQ883690	DQ986889
Maronea constans*	(1) Castello and Campagnolo 15972 (TBS; LSU). (2) China, Sipman 50094 (B; mtSSU)	–	–	AY640956	EF659771
*Meridianelia maccarthyana*	Australia, Tasmania, *Kantvilas* 752/03 (F)	–	–	–	FJ763185
*Ophioparma handelii*	China, Tibet, *Obermayer* 5135 (O L-168529)	–	KF360413	–	–
*Ophioparma lapponica*	Norway, *Timdal* 12353 (O L-170853)	divaricatic acid, usnic acid	KF360414	–	KF360443
*Ophioparma ventosa* 1	Norway, *Haugan* 7615 (O L-151477)	–	KF360415	KF360474	KF360444
*Ophioparma ventosa* 2	Norway, *Bjelland* 60 (BG)	–	AY011013	AY853380	AY853331
*Umbilicaria africana*	Peru, *Hestmark* 5081B (O)	–	HM161482	HM161545	HM161572
*Umbilicaria aprina*	Bolivia, *Hestmark* 5030B (O)	–	HM161483	HM161514	HM161573
*Umbilicaria crustulosa*	Norway, *Hestmark* 9017 (O)	–	HM161496	HM161590	HM161612
Umbilicaria proboscidea*	(1) U.K., E:DNA:EDNA10-00739 (ITS). (2) *Lumbsch* 12165b (F; LSU, mtSSU)	–	FR799305	AY300870	AY300920
*Umbilicaria spodochroa*	Norway, *Hestmark* 3201 (DUKE 47907)	–	HM161481	DQ986773	DQ986815
*Xylopsora canopeorum*	U.S.A., California, *Reese Næsborg* 1544 (NY)	friesiic acid	–	–	–
*Xylopsora canopeorum* 1	U.S.A., California, *Reese Næsborg* 1522 (JEPS, holotype)	friesiic acid, thamnolic acid	**MG309307**	–	**MG309311**
*Xylopsora canopeorum* 2	U.S.A., California, *Reese Næsborg* 1707 (O 1316)	friesiic acid	**MG309309**	–	–
*Xylopsora canopeorum* 3	U.S.A., California, *Reese Næsborg* 1597 (O 1315)	friesiic acid	**MG309308**	–	**MG309312**
*Xylopsora canopeorum* 4	U.S.A., California, *Reese Næsborg* 1775 (JEPS)	friesiic acid	**MG309310**	–	**MG309313**
*Xylopsora caradocensis* 1	Norway, *Timdal* 2410 (O L-32967)	friesiic acid	KF360383	–	–
*Xylopsora caradocensis* 2	Sweden, *Westling* s.n. (S L-53582)	–	KF360384	–	–
*Xylopsora caradocensis* 3	Sweden, *Odelvik* 599 (S L-29227)	–	KF360385	–	KF360425
*Xylopsora friesii* 1 - cf.	Norway, *Timdal* 11029 (O L-158541)	friesiic acid	KF360388	KF360459	KF360428
*Xylopsora friesii* 2	Norway, *Breili* L3615 (O L-167185)	friesiic acid	KF360389	KF360460	KF360429
*Xylopsora friesii* 3	Sweden, *Wedin* 7139 (UPS)	–	–	AY853372	AY853324
*Xylopsora friesii* 4	Norway, *Timdal* 1055 (O L-56480)	friesiic acid	KF360390	–	–

New sequences are indicated by accession numbers in bold. * DNA sequences obtained from different vouchers; checked for gene-tree incongruence prior to concatenation. ** DNA sequences shorter that 200 bp and therefore not accepted for submission to GenBank. See Bendiksby and Timdal (2013) for sequences.

The four accessions of the tentatively new species group with significant support and showed themselves as sister to a clade consisting of three accessions of *Xylopsora friesii* (2, 3 and 4; Fig. 1). Four characters varied amongst the four accessions of the tentatively new species (three in the nrITS and one in mtSSU), none of which were parsimoniously informative within the group. Xylopsora cf friesii 1 differed from *X. friesii* 2, 3 and 4 in eight characters in the nrLSU, at least four in the mtSSU and at least 16 in the nrITS. The MP analyses supported *Elixia* as monophyletic (JK MSAmanual = 94 %; JK MSAsate = 87 %) with *Meridianelia* as sister (JK MSAmanual = 95 %; JK MSAsate = 93 %). In the ML and BI analyses, *Elixia* monophyly was not supported, as *Meridianelia* grouped with accessions of *E. flexella* and *Elixia* sp. and excluded *Elixia cretica*. This topology was not significantly supported by any analyses. The sister-relation between the Elixiaceae and the Ophioparmaceae was significantly supported only by Bayesian PP (Fig. 1).

**Table 2.** Tree statistics from various phylogenetic analyses (MP, ML, BI) of the MSAmanual and MSAsate alignments.

	Locus	Taxa	AL-length	PIC*	MPTs	MP Tree-length	CI	RI	RC	SATe ML score	BI ASDSF**	BI burn-in
MSAmanual	nrLSU	32	865	111								
	mtSSU	35	787	160								
	nrITS	45	505	164								
	concat	54	2157	435	1220	1479	60	79	46.6	-8926.214413	0.003623	21.7%
MSAsate	nrLSU	32	865	111								
	mtSSU	35	793	162								
	nrITS	45	509	174								
	concat	54	2167	447	10	1485	59	79	46	-9030.310897	0.005327	13.7%

* ingroup ** at termination (i.e. generation no 6 mill)

## Discussion

Forest canopies in general are relatively understudied because accessing the tree crowns requires technical expertise and equipment (Lowman et al. 2012). Therefore, the potential for encountering new species is relatively high compared to more easily accessible forest floor environments. The new species presented here, *Xylopsora canopeorum*, has so far only been registered from the crowns of large coast redwood trees, but other similar habitats, like the fibrous bark of other large members of Cupressaceae, should be explored for the species. The collected *Xylopsora canopeorum* specimens occurred on stable bark surfaces of old, large redwood trees together with several species previously classified in the genus *Hypocenomyce* (e.g. *Carbonicola anthracophila*, *Fulgidea oligospora*, *F. sierrae*, and *H. scalaris*), which was recently shown to be highly polyphyletic (Bendiksby and Timdal 2013). Together, these species covered a substantial portion of the trunk surface.

### Molecular analyses

Automatic alignment by SATé-II differed only slightly from the manually aligned dataset. Only areas with ambiguous alignment solutions varied between the manually aligned multi-locus alignment (MSAmanual) and the one aligned automatically (MSAsate). Moreover, the two alignments rendered highly similar topologies when analysed using the same algorithm. MSAsate contained slightly more parsimony phylogenetic information and produced fewer MPTs. Although the different algorithms produced variously resolved trees, the same significantly supported clades were present in all output trees. This suggests significant time-savings by using SATé-II and software of similar quality for both automated alignment and phylogenetic analyses.

As expected, the overall tree-topology (Fig. 1) largely corresponded to previous findings (Bendiksby and Timdal 2013: fig. 2A), the only exception being that *Elixia* monophyly was not supported by the BI and ML analyses and supported only by MP (JK MSAmanual = 94 %; JK MSAsate = 87 %). The grouping of *Meridianelia* with accessions of *E. flexella* and *Elixia* sp., however, was not significantly supported by any analyses (Fig. 1) and was not considered of taxonomical significance. More importantly, monophyly of the four newly included accessions was significantly supported regardless of alignments or analyses algorithm. Likewise, this clade’s sister relation to *X. friesii* was significantly supported. The low and non-informative genetic variation between the four newly included accessions strongly suggests they belong to a single species. The specimen Xylopsora cf friesii 1, on the other hand, differed significantly from *X. friesii* 2, 3 and 4. It is hypothesised that X. cf. friesii 1 represents a species distinct from *X. friesii*, but more material will need to be studied prior to drawing additional taxonomic conclusions.

## Taxonomy

### Xylopsora canopeorum

Taxon classificationFungiLecanoromycetesUmbilicariaceae

Timdal, Reese Næsborg & Bendiksby sp. nov.

MB823500

Fig. 2

#### Diagnosis.

The species differs from *X. caradocensis* and *X. friesii* mainly in forming more minute, coralloid and sometimes, sorediate squamules and sometimes (the holotype) in containing thamnolic acid in addition to friesiic acid; it also differs from the former in having shorter, non-septate ascospores.

#### Type.

USA, California, Santa Cruz Co., 75 m E of North Escape Road, 125 m S of the third gate on North Escape Road in Big Basin Redwoods State Park, 37°10'46"N, 122°12'58"W, 341 m alt., on bark of main trunk more than 100 cm diameter, from the upper trunk of old *Sequoia sempervirens* in old-growth redwood forest, fall (autumn) 2015, R. Reese Næsborg 1522 (JEPS, holotype [TLC: friesiic acid (major), thamnolic acid (submajor); GenBank: MG309307 (ITS), MG309311 (mtSSU)]).

#### Description.

Thallus crustose to squamulose; individual squamules up to 0.5 mm diam. but often soon breaking up into a coralloid crust, adnate when young, later ascending and more or less geotropically imbricate; soralia occurring patchily, labriform, bluish; upper surface greyish-green to medium brown, dull; margin crenulate or incised, concolorous with upper surface. Upper cortex up to 15 μm thick but mostly poorly defined. Apothecia common, up to 0.6 mm diam., plane, black, epruinose, egyrose; margin remaining prominent, entire or flexuose; proper exciple composed of closely conglutinated hyphae, olivaceous brown in inner part, brownish black in the rim, not containing crystals, K–, N–; hymenium ca. 50 µm high, pale olivaceous brown; hypothecium pale olivaceous brown; epihymenium dark reddish brown, not containing crystals, K–, N–; paraphyses ca. 2 µm thick, simple, without swelling or pigment cap in apical cell; ascus clavate, ca. 30 µm tall, with a thin, evenly amyloid tholus and covered by an amyloid cap, with orange pigment in the cytoplasm when young. Ascospores ellipsoid, simple, hyaline, with orange pigment in the cytoplasm when young, 4–7 × 2.5–4.5 μm (n = 20, from holotype). Pycnidia not seen.

#### Chemistry.

Friesiic acid (major) and thamnolic acid (absent to submajor). Thallus PD– or PD+ yellow, K– or K+ yellow, C–, UV+ bluish white.

#### Distribution.

Specimens were collected from central coastal California in Big Basin Redwoods State Park (37.1°N, 11 km from the Pacific Ocean) and Armstrong Redwoods State Natural Reserve (38.3°N, 18 km from the Pacific Ocean).

#### Ecology.

*Xylopsora canopeorum* was observed on coarse, fibrous bark and occasionally on charred bark between 5 and 75 m above ground level along the trunks of large coast redwood trees in old-growth redwood forests. The species commonly co-occurred with *Carbonicola anthracophila*, *Fulgidea oligospora*, *F. sierrae*, *Hertelidea botryosa* and *Hypocenomyce scalaris*, which together covered substantial portions of the trunk surface. *Xylopsora canopeorum* appeared to have an affinity for old and stable bark surfaces on the main trunks of large redwood trees.

#### Etymology.

The specific epithet “*canopeorum*” refers to the habitat in which the species was encountered ¾ in the canopy of old-growth redwood forests.

#### Remarks.

The species differs from *X. caradocensis* and *X. friesii* morphologically by forming more minute squamules (less than 0.5 mm diam.) which soon break up into a coralloid crust and sometimes into soralia. In *X. caradocensis* and *X. friesii*, the squamules are up to 1.0 (–1.5) mm diam. and always esorediate. In the former, the squamules are bullate or irregularly ascending; in the latter more or less plane, adnate or somewhat ascending (Timdal 1984). In *X. caradocensis*, the ascospores are longer (6.5–14 × 2–4 μm) than those of *X. canopeorum* and often 1- or 3-septate; in *X. friesii*, the ascospores hardly differ (4.5–7.5 × 2.5–3.5 μm) from those of *X. canopeorum*. *Xylopsora caradocensis* and *X. friesii* contain friesiic acid only (Timdal 1984, as “friesii unknown”).

In the current Californian lichen checklist (Tucker 2014), *Lecidea xanthococcoides* Zahlbr. is the only species unknown to the authors that could be assumed to be an earlier name for *X. canopeorum*. That species was described from conifer trunks at 1700 m alt. in the San Bernardino Mountains, i.e. in an area and habitat where *X. canopeorum* possibly can occur. The holotype (H.E. Hasse 705) was not found in W upon enquiry. Details in the original description (Zahlbruckner 1900) indicate that it is a different species, however – Apothecia becoming convex and immarginate, hymenium 160–180 μm high and ascospores 12–15 × 5.5–6 μm.

#### Additional specimens examined.

USA. California. *Santa Cruz Co.*: label data as for holotype, R. Reese Næsborg 1544 (NY); 800 m WNW of North Escape Road up Rodgers Creek in Big Basin Redwoods State Park, 37°11'44"N, 122°13'34"W, 403 m alt., on bark of branch less than 50 cm diameter in the lower crown of an old *Sequoia sempervirens* tree in an old-growth redwood forest, spring 2015, R. Reese Næsborg 1597 (O L-1315); 400 m E of North Escape Road along Sequoia Trail in Big Basin Redwoods State Park, 37°11'13"N, 122°12'54"W, 422 m alt., on bark of trunk more than 100 cm diameter in the upper trunk of an old *Sequoia sempervirens* tree in an old-growth redwood forest, Fall 2015, R. Reese Næsborg 1707 (O L-1316). *Sonoma Co.*: 50 m SW of Colonel Armstrong Tree parking area in Armstrong State Natural Reserve, 38°32'13"N, 123°00'29"W, 49 m alt., on bark from the upper trunk of an old *Sequoia sempervirens* tree in an old-growth redwood forest, fall 2015, R. Reese Næsborg 1775 (JEPS).

**Figure 2.**
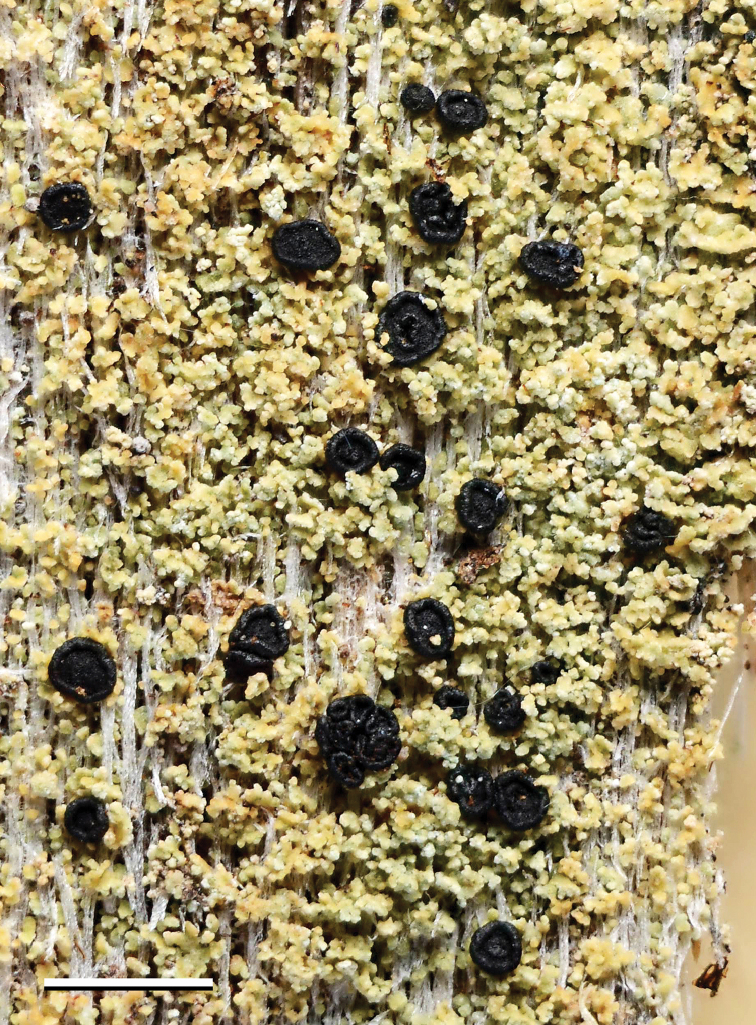
*Xylopsora canopeorum*, holotype. Scale bar: 1 mm.

## Supplementary Material

XML Treatment for Xylopsora canopeorum

## Supplementary materials

Supplementary material 1 MSAmanual.nex Link: https://doi.org/10.3897/mycokeys.30.22271.suppl1 Data type: Sequence alignment Explanation note: A manually aligned concatenated multiple sequence alignment comprising three genetic regions (nrLSU, mtSSU and nrITS) and a subset of 54 accessions representing the Elixiaceae, the Fuscideaceae, the Ophioparmaceae, and the Umbilicariaceae. This is an open access article distributed under the terms of the Creative Commons Attribution License (CC BY 4.0), which permits unrestricted use, distribution, and reproduction in any medium, provided the original author and source are credited.Mika Bendiksby, Rikke Reese Næsborg, Einar Timdal

Supplementary material 2 MSAsate.nex Link: https://doi.org/10.3897/mycokeys.30.22271.suppl2 Data type: Sequence alignment Explanation note: An automatically aligned (using the software SATé-II version 2.2.7) concatenated multiple sequence alignment comprising three genetic regions (nrLSU, mtSSU and nrITS) and a subset of 54 accessions representing the Elixiaceae, the Fuscideaceae, the Ophioparmaceae, and the Umbilicariaceae. This is an open access article distributed under the terms of the Creative Commons Attribution License (CC BY 4.0), which permits unrestricted use, distribution, and reproduction in any medium, provided the original author and source are credited.Mika Bendiksby, Rikke Reese Næsborg, Einar Timdal
